# The effect of GeoGebra integrated instruction on students’ learning of the quadratic function concept

**DOI:** 10.12688/f1000research.163113.1

**Published:** 2025-07-07

**Authors:** Ashebir Sidelil Sebsibe, Neja Muzea Abdella

**Affiliations:** 1 Mathematics, Wachemo University, Hosaena, Central Ethiopia, 667, Ethiopia; 2 Mathematics, Worabe Polytechnic College, Worable, South, 667, Ethiopia

**Keywords:** GeoGebra Software, Quadratic Function, Motivation to learn, Mathematics

## Abstract

**Background:**

GeoGebra® (a registered trademark of the International GeoGebra Institute) is dynamic, user-friendly, and open-source software that effectively connects geometry and algebra. By reducing abstraction and procedural demands, it facilitates students’ conceptual understanding. This study investigated the impact of GeoGebra integrated mathematics instruction on Grade 9 students’ achievement, conceptual understanding, and motivation regarding quadratic functions.

**Methods:**

A quasi-experimental design with non-equivalent control groups was employed, involving 42 students in the experimental group and 45 in the control group, drawn from different intact classrooms in Worabe Town Administration, Ethiopia. The experimental group received instruction using GeoGebra, while the control group followed traditional teaching methods. The intervention was grounded in social constructivist theory. Data collection instruments included pre- and post-tests for mathematical achievement and conceptual understanding, as well as a motivation questionnaire. Statistical analysis was conducted using SPSS.

**Results:**

The experimental group showed statistically significant improvement in both conceptual understanding and problem-solving performance compared to the control group (p < 0.05). Additionally, students in the GeoGebra-integrated instruction group reported significantly higher levels of motivation toward learning quadratic functions.

**Conclusions:**

GeoGebra-integrated instruction has the potential to improve student achievement, reduce time spent on routine calculations, and address challenges related to multiple representations in mathematics. Furthermore, it enhances student motivation and engagement with critical mathematical concepts. These findings support the inclusion of dynamic mathematical software like GeoGebra in secondary mathematics instruction, especially for topics requiring visualization and symbolic reasoning.

## Introduction

The level of success that students achieve in mathematics is seen as a key factor in determining not only the future of the individual learner but also the future of society as a whole. There is a global consensus that proficiency in mathematics reflects the country’s education system, which is closely linked to the nations prosperity. ^1– 3^ Success in the subject fosters critical thinking and problem-solving skills that are crucial in the 21st-century job market. ^4^

The function is a fundamental concept in mathematics that links to various ideas in both basic and advanced areas, such as calculus and geometry. ^5– 7^ Understanding function concepts in particular and the quadratic function concept in particular significantly influences students’ learning, especially when they progress to higher levels of education. ^8^ The ability of students to comprehend quadratic functions and transition between algebraic expressions and graphical representations is crucial. This proficiency paves the way for tackling more complex functions. Students begin applying this concept in high school science and mathematics, and it carries through their college studies. Many individuals utilize this concept to analyze real-world issues. ^9– 11^

On the contrary, several studies (for example, ^10, 12– 15^) have shown that students often struggle with understanding the concepts related to quadratic functions. Issues such as difficulty in relating the two variables (i.e., dependent and independent), a lack of comprehension regarding real-life applications, and challenges in switching between different forms of representation (symbolic, graphical, and numerical) including reverse thinking and interpreting the parameters of a quadratic function are common. Additionally, students often misapply properties of linear functions to quadratic functions, which add to their difficulties. ^10, 13, 15^ Therefore, it is essential to further explore the reasons behind the challenges many students face when solving problems related to this topic. ^16^

### Problem statement

One of the major challenges facing the Ethiopian education system is the low performance and lack of motivation among students in mathematics. ^17– 20^ Notes that students exhibit low levels of mathematical competency and their interest also shows a declining trend over time. For example, in the university entrance examination, the average scores of students dropped from 41.11 in 2016/17 to 38.55 in 2019/20, highlighting the inadequate mathematics skills of students entering university.

In the study area under discussion, the students’ performance was arguably worse. The Annual Abstract Statistics report by the Silte Zone Education Department (SZED) stated that the percentage of students who scored at least half on the mathematics entrance examination dropped from 18% to 12% in subsequent years. These findings indicate that there is an urgent need to study students’ performance and motivation for mathematics. Consequently, the main areas where students require assistance in understanding are functions and graphs, which are fundamental concepts in advanced mathematics. ^21^ Argue that the school, teaching methodology, and the participation of students greatly affect learning outcomes. Thus, we need to investigate better practices that engage students as active participants in the teaching-learning process. In that sense, ^17, 22^ emphasized the design of alternative methods for teaching to improve student performance and motivation in science and mathematics instruction. Instructors should give thought to current student expectations, the country’s development plan, contemporary learning theories, and the role of technology in education.

Ethiopia General Education Curriculum Framework (EGECF) acknowledges that the 21st-century society is characterized by modern advancements in Information and Communication Technology (ICT), digital learning, and any components therewith. Thus, digital literacy shall be part of the general curriculum and applicable at different levels, for which attainment shall be ensured for all learners. ^23^ As mentioned by, ^24^ since technology is booming with a very short half-life, the traditional instructional approaches are not suitable to attain the 21 ^st^-century educational objectives that demand an active learning process or student-centered learning approach.

In acknowledgment of the benefits of being successful in mathematics, the new EGECF indicated developing a curriculum that emphasizes the significance of mathematics has added value to the teaching of other subjects and hence to the advancement of science and technology. Besides, the curriculum document emphasized the technology-integrated mathematics instruction to smooth the process of teaching-learning for the benefit of learners. Several research studies (for instance, ^1, 25– 28^) have suggested levels of technology-integrated instruction either to improve student performance and interest in mathematics or to motivate learners and overcome observed problems.

The driving force for this study is the nationally recognized concerns, i.e. the current practice does not lead to the realization of the country’s development plan and educational goals Therefore, improved performance and motivation require the development of a different approach in science and mathematics instructions. ^22, 29^

There are different mathematical software’s designed to develop student learning attitudes and improving achievement: GeoGebra, **Mathematica ^®^**, and MATLAB **^®^** are some of them. These tools are proprietary software developed by the International GeoGebra Institute, Wolfram Research, and MathWorks, respectively.

GeoGebra, given its nature and advancement, can be regarded as the most important mathematical software for it is intended to show two pillars of representation in mathematics; geometric and algebraic, on the same computer screen. Also, it is intentionally designed for educational purposes and enables students to improve their performance and interest in studying the subject. ^30^

According to, ^24^ GeoGebra is not yet widely used to teach mathematics in Ethiopia. What’s more, it’s easy to use; choices of representation that can be made with one’s preferred colors and includes a slider/“Dynamic Movement” types. ^28, 31^ Add that it would be beneficial to find out what impact GeoGebra can have on the acquisition of pedagogical knowledge deemed necessary for effective use of GeoGebra by the learners. Today’s digital technology practice also provides evidence that we should act accordingly.

Thus, this study aims to assess the efficacy that GeoGebra software integrated mathematics teaching has on student achievement and motivation compared to conventional instruction. In doing so, the study tried to answer the following research question: what effect does the use of Geogebra software integrated instruction has on students learning the quadratic function concept in terms of achievement, concept acquisition, and motivation?

### Theoretical framework of the study

With the understanding that computers can influence learning, this study is grounded in the Social constructivism theory. It investigates how students utilize virtual manipulatives, specifically the GeoGebra applets, to enhance their learning outcomes and motivation regarding the concept of quadratic functions.

Social Constructivism Approach (SCA): SCA builds on Vygotsky’s Zone of Proximal Development (ZPD) and consists of four levels that explain how students grasp a concept, with the teacher acting as a facilitator. The degree of facilitation varies based on the learner’s current level of understanding the concept under consideration. In this study, the integration of GeoGebra in instruction acts as a scaffolding tool, while peers function as a community that aids in concept formation. Teachers must be aware of their students’ prerequisite knowledge and cognitive levels to provide suitable assistance tailored to their needs at each ZPD level. ^32^ The use of GeoGebra applets was guided by the four-level model proposed by. ^33^

Level I: At this stage, the teacher is expected to support students as they tackle specific tasks. To prepare them for the next level, the teacher should consistently ask questions to gauge their understanding of fundamental concepts. ^32^ Support can come from the teacher or a more knowledgeable peer. ^33– 35^ This collaborative interaction benefits students of varying abilities. With ongoing practice and support, students can advance to Level II.

Level II: Here, students engage in specific tasks with less dependence on their teacher ^35^ and draw on their prior knowledge to complete tasks independently. ^36^ However, some tasks may still pose challenges for students to finish on their own ^37^ as students’ autonomy is not yet fully developed. ^38^ For instance, in the implementation of GeoGebra, at this level, they actively engage with GeoGebra applets and regularly request assistance from either the teacher or more capable colleagues for help.

At Level III, external support becomes less beneficial; students’ performance becomes internalized, developed, and automated. ^33^ This stage marks the transition from the ZPD to a more advanced level of competence for a specific task. ^32^

At Level IV, students begin to reverse their execution steps, leading to revisions of their work. They apply insights gained from past experiences organized through the ZPD. Lasting learning involves cycling through the same controlled ZPD stages, moving from assistance to self-sufficiency repeatedly to enhance untapped abilities. ^33^

### Overview of the GeoGebra software

GeoGebra, developed by Markus Hohenwarter, ^30, 39^ is an open-source dynamic mathematics program that seamlessly integrates algebra, statistics, calculus, and geometry into a single, user-friendly interface. The name GeoGebra reflects how its interface displays each mathematical object: once in the graphic (geometry) window and once in the algebra window. This software enables students to visualize mathematical processes and provides intuitive guidance. With its various tools, students can explore a wider range of function types by linking symbolic and visual representations. ^40^ The immediate changes that occur when an object is modified in one of these windows enhance the learner’s ability to recognize key cognitive relationships. While participants in this study utilize this software for various applications, they specifically engage with lessons created using it.

**Figure 1.**
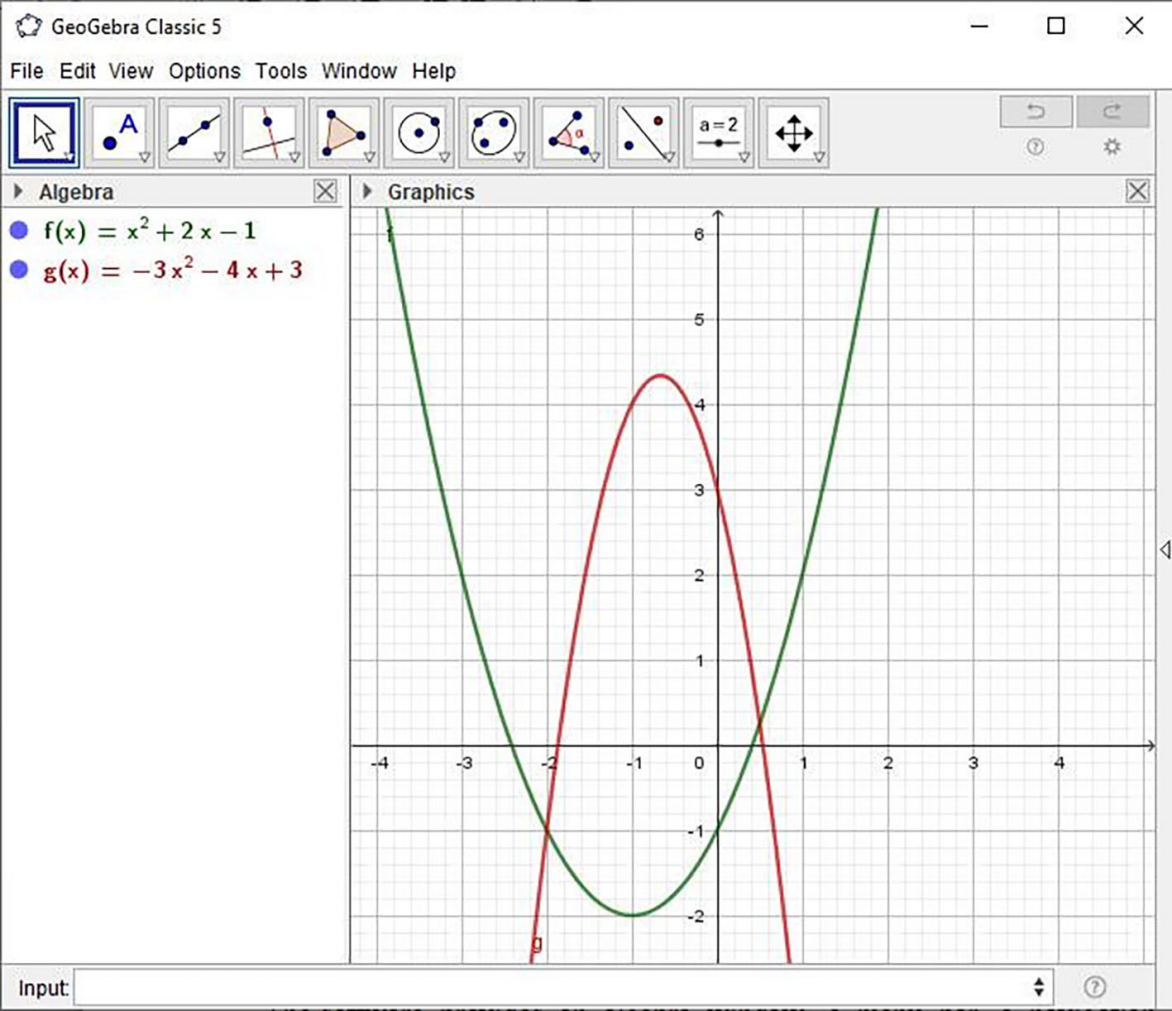
Screenshot from a GeoGebra Window.

Figure 1 displays a screenshot of the GeoGebra window, illustrating the connection between geometry and algebra. As shown in the figure, multiple functions can be easily demonstrated on the same screen. The software includes an algebra window, a menu bar, a navigation bar, an input field, a geometry window or working area, and a toolbar. Although its primary focus is on teaching geometry, it also offers excellent features for teaching algebra, particularly in the areas of functions and graphs.

Functions can be defined algebraically and then modified dynamically. For instance, when an equation is entered, the corresponding graph is generated immediately in the geometry area, while the algebraic representation appears in the algebra window at the same time. This tool is user-friendly, reduces the effort needed to manage processes, and offers options for representation with preferred colors and types of slider. ^31^ These features help minimize the abstraction needed to grasp concepts, which is a significant challenge for students learning about quadratic functions. ^12^

The impact of GeoGebra on students’ achievement and motivation has been examined in various contexts. Some notable benefits include graphic displays that clarify abstract concepts and boost motivation to learn, instant feedback that can validate or challenge a student’s actions, and the enhancement of both domain-specific knowledge and digital skills. ^41– 43^ These studies demonstrate that instruction integrated with GeoGebra leads to improved learning outcomes for students.

## Method and materials

The study utilized a quasi-experimental design with a non-equivalent comparison group, employing a quantitative approach. This choice was made because the samples were drawn from two different schools, making it impractical to randomly assign students to groups due to varying class schedules and other managerial, disciplinary, and ethical considerations. As noted by, ^44^ a quasi-experiment is a robust method for evaluating the causal impact of an intervention on its intended audience.

The study took place in Worabe Town Administration (WTA), located in the Siltie Zone of Ethiopia. The focus of this research was on all Grade 9 students within the Town Administration. According to the 2020’s SZED Annual Statistics, WTA has consistently underperformed in the grade 12 entrance examination compared to other Woredas and town administrations in the Zone over several years. The Town Administration, which includes four secondary schools, was selected as the target for this study. These four schools have a combined total of 625 9th-grade students. However, two of the schools were excluded for specific reasons: one was newly established at the time of the study and lacked a historical background, while the other was a special boarding school with unique circumstances. Therefore, the remaining two senior schools, which together have 473 9th-grade students (210 male and 263 female) across nine sections or classrooms, were the focus of the research.

Students from one classroom in each of the two different schools were selected, and they were randomly assigned to groups (control and experimental). The control group (N = 45) was taught using the traditional method (chalk-and-talk), while the experimental group (N = 42) received instruction that integrated GeoGebra in the school’s computer lab. With a total sample size of 87, which represents about 18.4% of the population, this is considered a reasonable sample size. ^45^

Both groups took the same tests, which included a pre-test and a post-test, along with a motivation attribute questionnaire. Initially, a pre-test with 18 items was given to both groups to assess their comparability regarding function before the intervention began. After two weeks of instruction (ten days), a post-test was conducted, featuring 27 items of various types categorized into three outcomes based on the concepts they aimed to address. The number of items in the post-test corresponding to each outcome is detailed in Table 1.

**Table 1.** Post-test items.

Outcome	Concepts	Items
1	Interpret and use the Vertex Form of a Quadratic Function (IUVFQF)	1, 3, 4, 6, 8, 10, 11, & 12
**2**	Interpret and use the Standard Form of a Quadratic Function (IUSFQF)	2, 7, 9.1, 9.2, 13, 14.1, 14.4, 14.5, 15.1, 15.3, & 15.4
**3**	Shifting Rules of parent function i.e. f *(x) = x ^2^* to Sketch Graph of other Quadratic Functions (SR-SGQF)	5, 8, 14.2, 14.3, 14.4, 14.5, 14.6, 14.7, 15.2, 15.5, 15.6

The test items were created using national examination booklets from previous years, grade 9 textbooks, and model exams from a specific secondary school in the town. They were chosen based on the grade 9 mathematics syllabuses and the minimum learning competencies outlined in it. Five experienced mathematics teachers and two curriculum experts evaluated and critiqued the selected items. Additionally, a pilot test was conducted with 40 randomly chosen 9th-grade students from a non-participating special secondary school, and the reliability and validity of the items were thoroughly analysed. All necessary modifications were made before the final administration.

To answer the motivation question, a questionnaire of two parts where administered. The first part, a six-item questionnaire of motivation factors relevant to the teaching and learning process, and the second part, an eight-item questionnaire of perception towards the GeoGebra integrated instruction, were included. While these motivational attributes include class participation, focus during lessons, enjoyment of class activities, self-confidence, mastery of concepts, and preference for the instructional method, the perception attributes include students’ level of interest in the lesson environment, the opportunity to reduce misconceptions, and enhance achievement. The questionnaire aimed to analyse students’ learning motivation in GeoGebra integrated instruction compared to traditional methods, and was prepared based on the information obtained from the literature.

### The intervention

**Figure 2.**
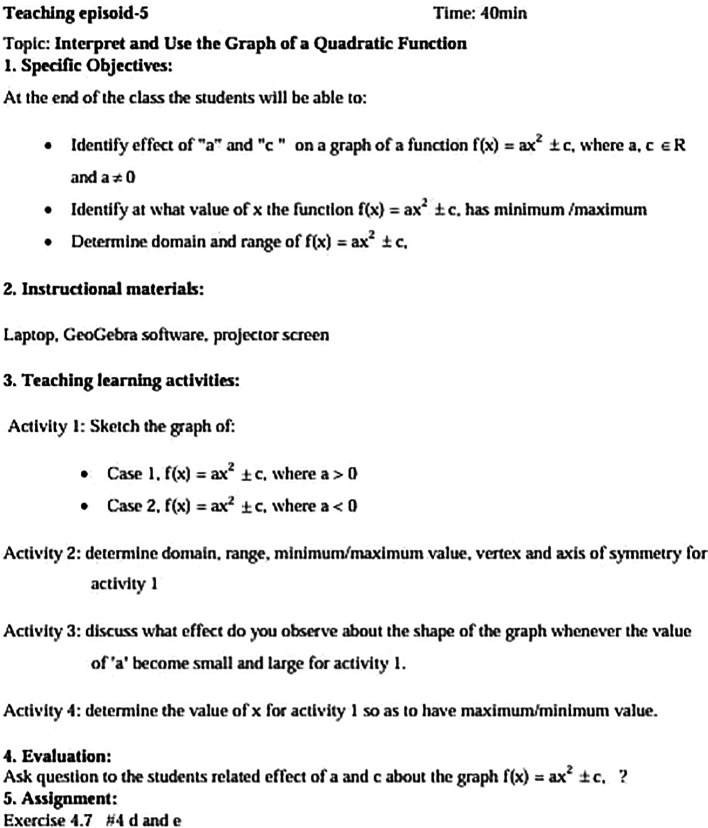
An example of the teaching episode.

For the intervention, the control group received instruction based on traditional methods, while the experimental group was taught using GeoGebra software to explore concepts across three themes. After a two-day introduction to using GeoGebra (which corresponds to level I of the theoretical framework), instruction continued with the help of an overhead projector. The second researcher delivered ten different lesson episodes to both groups. Each episode of the experimental group was designed to address the three concepts as identified in Table 1. Figure 2 illustrates one such episode.

In the computer lab, twenty-one computers were set up for the experimental group in pairs to encourage collaboration. The instruction included explanations, examples, demonstration activities, two summary worksheets, and presentation tasks, all carefully designed to meet the various levels of the theoretical framework. The researcher took on a facilitating role, correcting and checking the tasks saved in folders named after each student on their desktop. In contrast, the control group completed the same tasks but did not use the GeoGebra software.

Through the manipulation of the GeoGebra applets, the participants got a chance to see the effect of the variation of one variable on the other variable. Besides, the learners were free to learn according to their learning preference as suggested in the selected theoretical frameworks, and left free to communicate with their colleagues through their ways of thinking and language.

### Theoretical framework integration

GeoGebra was employed as a scaffolding tool. The teacher (in this case, the second author) used it to help students letting them move through the four stages of ZPD: at Level I, the teacher showed students how to use GeoGebra and walked them through basic exercises to make sure they got the main ideas and their implementation. At Level II, students started to work with quadratic functions on their own using GeoGebra but asked for help when they needed it. At Level III, students were proficient (and those who have a gap consult their peers or the teacher) in the concepts, seeing and manipulating functions using GeoGebra independently of the teacher. Finally, at Level IV, students strengthened what they learned through practice and used it to tackle harder problems from a worksheet and the textbook.

### Data analysis

Traditional descriptive statistical (frequency count, percentage, mean, and standard deviation) as well as inferential statistical techniques (independent sample test selected based on the assumptions the data satisfied) were used. The analysis was focused on checking whether there is a significant difference or not in students’ achievement and motivation to learn between the groups. The hypotheses corresponding to each research question were tested at a 95% confidence interval using a two tailed test.

### Ethical issues

In the study, the researchers endeavored to satisfy all admissible requirements for safeguarding and guaranteeing the rights of all those involved. The research obtained permission from Wachemo University Department of Mathematics DGC (Department Graduate Committee) on 27 March 2013 (Date 18/7/2015E.C, Ref. No WCU/CNCS/Math/0145/2015), and the researchers accepted the approval letter on April 2023 from the department. The university letter was submitted to SZED to obtain letters to the sample schools. The letters were submitted to the relevant school principals. The students were also informed in full about their involvement in the study and of their participant rights. Besides, the students signed a written consent form prepared in English and translated to the local language (Amharic) to express their agreement. The consent form was part of the proposal approved by the DGC. The study was conducted from May 8 to May 26, 2023. The participants’ responses were coded and their identities were not revealed in the study report.

## Findings and discussion

### Pre-test result

The pre-test results indicated that the average (M) and standard deviation (SD) of the control group students’ achievement (M = 10.36; SD = 2.693) were nearly identical to those of the experimental group students (M = 10.36; SD = 3.399). Additionally, the T-test results confirmed that both groups had similar levels of prerequisite knowledge before the treatment (p-value 0.998 ≥ 0.05). Therefore, any differences observed in the understanding of quadratic functions after the intervention can be attributed to the treatment itself.

### Post-test result

**Effect of GeoGebra-integrated Instruction on Students’ Achievement**

While Table 3 provides a summary of the statistics for both groups, Table 4 presents the results of the independent sample t-test comparing the two groups. The findings in Table 3 indicate that the control group students had a lower achievement level (M = 17.60; SD = 4.614; SE = 0.688) compared to the experimental group students, who had a higher average achievement (M = 27.90; SD = 2.844; SE = 0.688).

According to the data in Table 4, Levene’s test shows a significant result (p = .00, which is less than 0.05). This indicates that there is a significant difference in variance. ^47^ In both scenarios, the outcome remains consistent, demonstrating a significant difference in the performance of the two groups based on their mean values (at an alpha level of 0.05, two-tailed probability), with the experimental group performing better. Therefore, we can conclude that the GeoGebra integrated instruction had a positive impact on students’ understanding of quadratic function concepts.

To strengthen the conclusion (to see magnitude of the effect), we have also calculated the effect size (r) of the result. Accordingly, we used the formula $r=\sqrt{\frac{t^{2}}{t^{2}+\mathrm{df}}}$ ^47^ where t and df as in Table 3. Thus, $r=\sqrt{\frac{{\left(-12.63\right)}^{2}}{{\left(-12.63\right)}^{2}+73.959}}$ = 0.83 which implies the effect is large (Cohen, 1992 as in ^47^).

**Effect of GeoGebra integrated instruction on students’ conceptual understanding**

**Figure 3.**
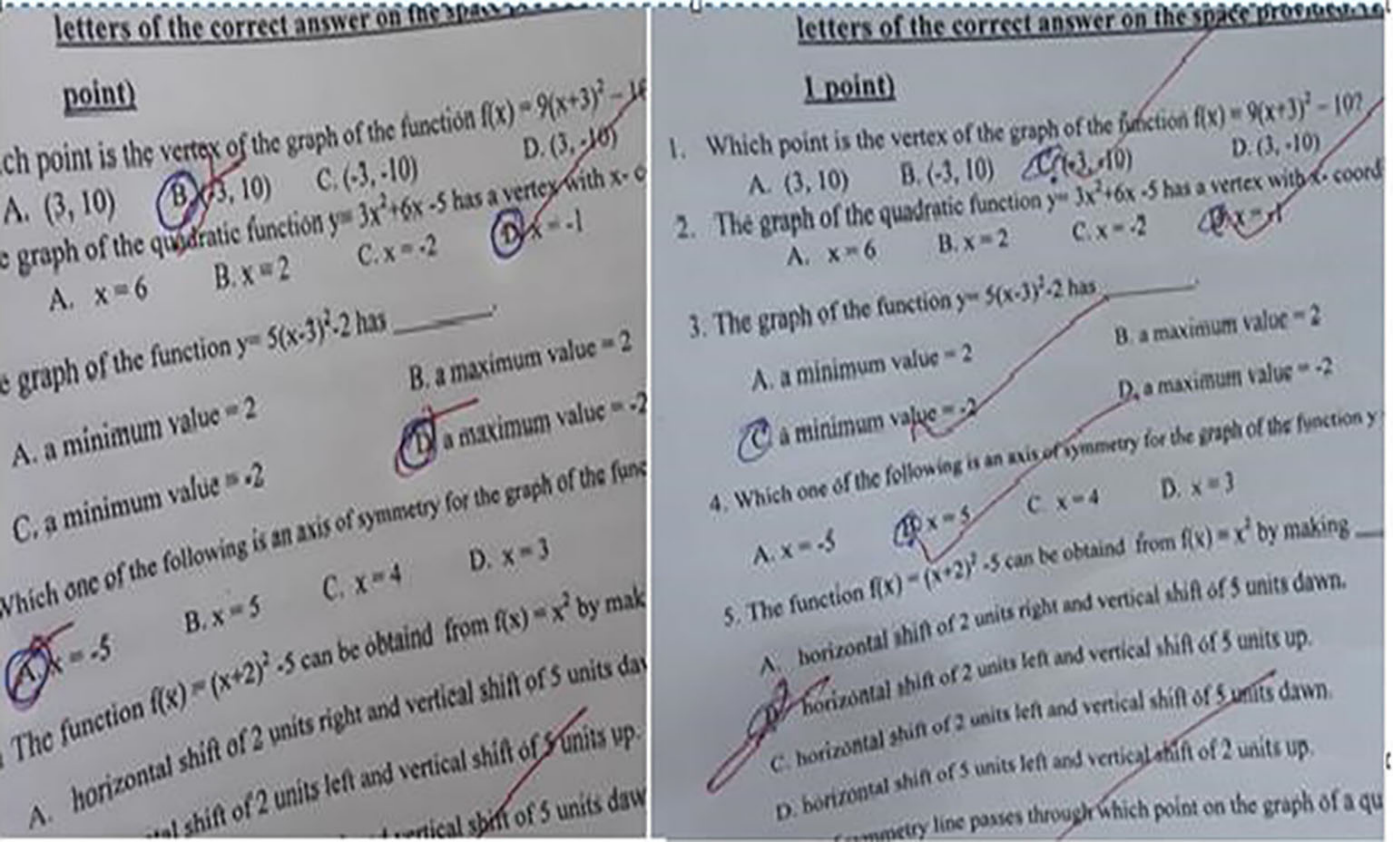
A scanned image of the works of students.

For items related to outcome 1, the overall percentage of correct responses for the control group was 59.45%, while the experimental group achieved 83.93%. This suggests that the intervention helped the experimental group better understand the IUVFQF concept. Additionally, the control group had a mean percentage of 69.23%, compared to 89.16% for the experimental group in answering items related to outcome 2. This further indicates that the experimental group outperformed the control group in grasping the IUSFQF concept. Figure 3 displays scanned images of students’ test scripts, with the left side representing the control group and the right side showing the experimental group, for items across all three outcomes.

**Figure 4.**
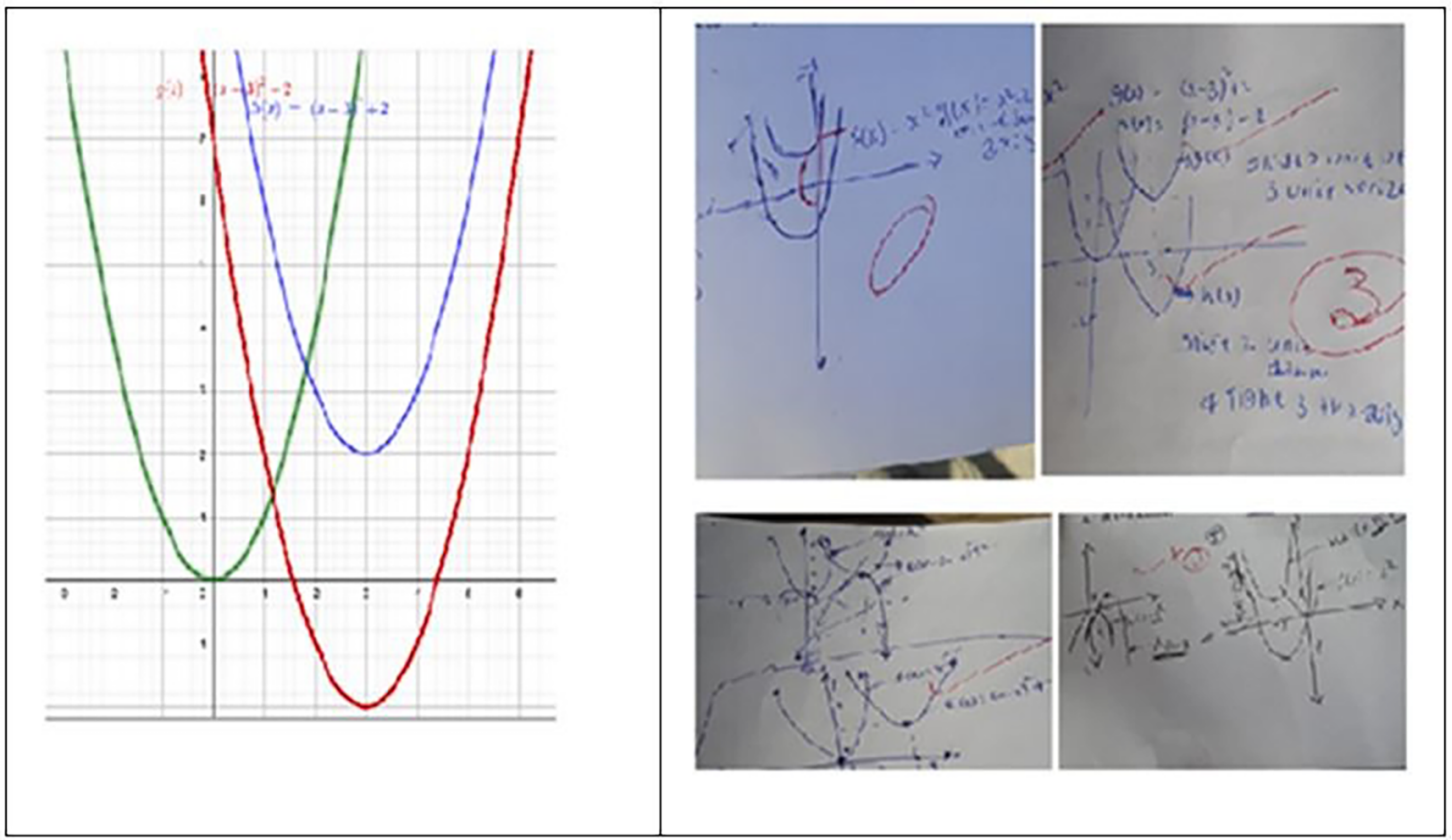
A scanned image of the works of students for items in outcome 3.

The students’ test results show that the control group achieved a mean percentage of 58.15% correct responses for items in outcome 3, while the experimental group had a significantly higher rate of 91.07%. This indicates that the experimental group outperformed the control group. Figure 4 displays scanned images of the students’ test scripts, with the left side representing the control group and the right side representing the experimental group for items in outcome 3.

**Figure 5.**
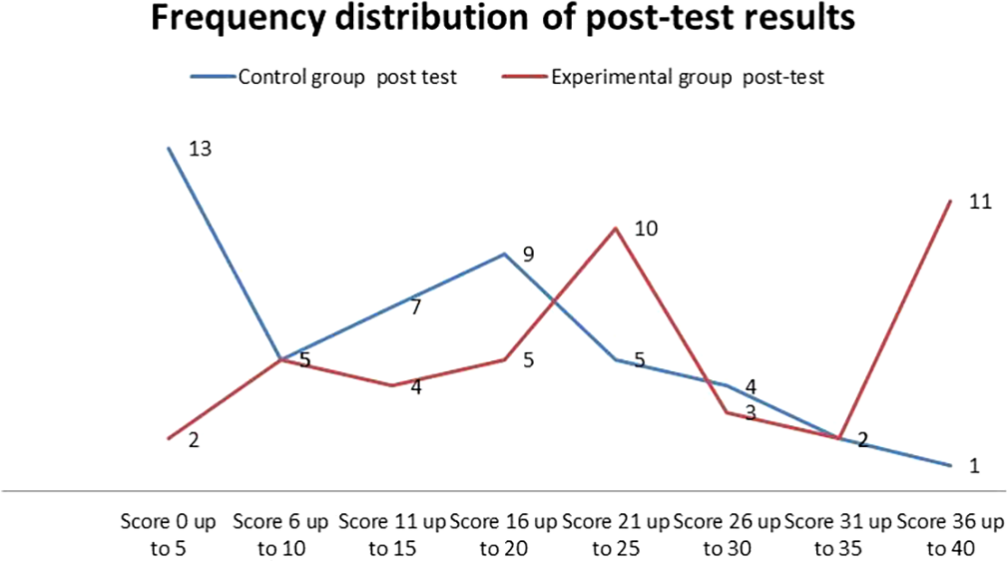
Frequency distribution of post-test results of the two groups.

The introduction of the applet significantly enhanced students’ understanding and eased the challenges associated with learning quadratic functions in grade 9. This improvement can be attributed to several factors, including a focus on grasping the conceptual aspects, a reduction in the effort needed for routine calculations, the ability to view multiple representations on a single screen, and the immediate feedback provided to verify the accuracy of their work through GeoGebra’s integrated instruction. Furthermore, the concepts and techniques learned by using applets like GeoGebra are lasting and can be seamlessly incorporated into students’ existing cognitive frameworks, making them more manageable. ^48^ Figure 5 illustrates the frequency distribution of the post-test results for the two groups.

In general, Figure 5 illustrates that the overall performance of students in the post-test differed between the two groups. Specifically, a greater number of students in the experimental group achieved higher scores, while more students in the control group scored at lower levels compared to those who performed at higher levels.

**Motivational effect of GeoGebra-integrated instruction**

**Figure 6.**
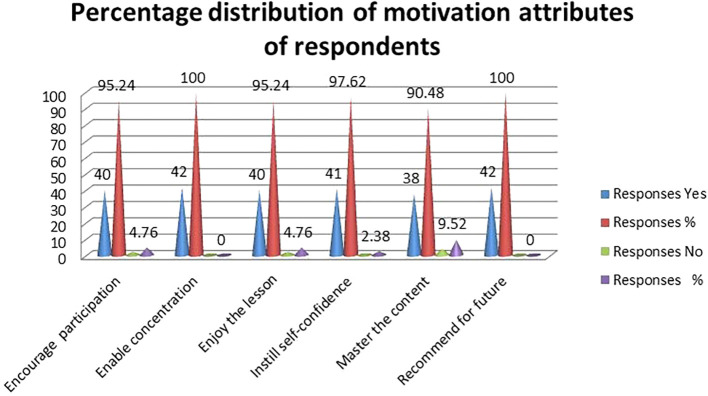
Percentage distribution of motivation attributes of respondents.

To explore the motivational impact of the GeoGebra integrated instruction, a questionnaire was developed that included six motivational attribute items and eight perception attribute items. Figure 6 illustrates the percentage distribution of these motivation attributes among the respondents based on the items in the motivation questionnaire.

As shown in Figure 6, the results indicated that out of the 42 students in the experimental group, 40 (95.24%) responded ‘Yes’ to the question: Are you motivated to learn about quadratic functions and their graphs? The experimental group demonstrated a strong motivation to learn these concepts. All students reported that the GeoGebra-integrated instruction encouraged their participation in the learning process, and 40 (95.24%) confirmed that it helped them maintain focus throughout the lesson.

Regarding the enjoyment of lessons, 41 (97.62%) of the students stated that learning about quadratic functions and their graphs using GeoGebra was very enjoyable. Additionally, 38 (90.48%) of the students who were asked if the instruction enabled them to answer every test item responded “yes,” indicating that the method effectively helped them grasp the concepts in the material. Furthermore, all respondents recommended the use of the GeoGebra integrated instructional approach for teaching concepts related to quadratic functions. Lastly, participants noted that their background in computer skills influenced their level of benefit, motivation, and satisfaction with the instruction.

Concerning the eight Likert scale perception questionnaire, the result is summarized in Table 2. As indicated in Table 2, all students have shown positive perception towards the GeoGebra-integrated instruction consistently through the eight items. Thus, despite the slight difference in ratings, the aggregated mean (M = 3.78, SD = .752) indicates that the respondents positively perceived the new experience. This is promising, if not absolutely a permit, to get the attention of students on hard concepts in particular and hard sciences in general.

**Table 2.** Independent sample t-test result of the pre-test.

Pre-test group	N	Mean	SD	t	df	P
Control	45	10.36	2.963			
Experimental	42	10.36	3.399	-0.002	85	0.998

**Table 3.** Group statistics of the post-test items.

	POSTTESTGROUP	N	Mean	Std. Deviation	Std. Error Mean
POSTTESTRESULT	Control Group	45	17.60	4.614	.688
	Experimental Group	42	27.90	2.844	.439

**Table 4.** Independent samples T-test for post-test items.

		Levene's Test for Equality of Variances		t-test for Equality of Means						
		F	Sig.	t	df	Sig. (2-tailed)	Mean Difference	Std. Error Difference	95% Confidence Interval of the Difference	
									Lower	Upper
Post-test Result	Equal variances assumed	14.324	.000	-12.433	85	.000	-10.305	.829	-11.953	-8.657
	Equal variances not assumed			-12.630	73.959	.000	-10.305	.816	-11.931	-8.679

**Table 5.** Students’ attitude towards the Geogebra-integrated instruction.

Attitude questionnaire items	M	SD
GeoGebra software creates interesting environment in the classroom (ጂኦጀብራ ሶፍትዌር ለመማር አስደሳች ሁኔታን ይፈጥራል፡፡)	3.810	0.707
I like GeoGebra software to use in teaching quadratic function in the classroom (ጂኦጀብራ ሶፍትዌርን በመጠቀም quadratic function መማር አስደስቶኛል፡፡)	3.810	0.740
GeoGebra software helps to reduce misconceptions while in learning quadratic function (ጂኦጀብራ ሶፍትዌር በመጠቀም በመማሬ የተሳሳተ አረዳድን እንድቀንስ አግዞኛል፡፡)	3.595	.912
GeoGebra software helps to increase mathematics achievement (ጂኦጀብራ ሶፍትዌር በመጠቀም በመማሬ የሂሳብ ውጤቴ አፈፃፀም እንዲጨምር አግዞኛል፡፡)	3.952	0.697
GeoGebra software helps me to improve quadratic function knowledge (ጂኦጀብራ ሶፍትዌር quadratic function እውቀቴ እንዲሻሻል ረድቶኛል፡፡)	3.810	.707
GeoGebra software visualizes quadratic function with its graph (ጂኦጀብራ ሶፍትዌር quadratic function ከግራፉ ጋር ያለውን ተዛምዶ በቀላሉ መመልከት እንድችል አድርጎኛል፡፡)	3.738	.767
Mathematics classroom becomes more interesting if teacher uses GeoGebra software. (መ/ራን ጂኦጀብራ ሶፍትዌር ተጠቅመው ቢያስተምሩ የሂሳብ መማር ማስተማሩ ስራ የበለጣ ሳቢ ሊሆን ይችላል፡፡)	3.738	.767
GeoGera based teaching helps me to remember for long time about quadratic function and its graph than the traditional instruction (ከወትሮው መንገድ ይልቅ ጂኦጀብራን መሰረት ያደረገ ት/ት ቢሰጥ ተማሪዎች ስለ quadratic function እና ግራፉ ለረጅም ጊዜ አይዘነጉትም፡፡)	3.786	.717
Average aggregate weighted M and SD	3.78	0.752

M = Mean; SD = Standard deviation.

Overall, the findings of this study suggest that using GeoGebra-integrated instruction is beneficial for teaching mathematical concepts at the secondary school level. The applet adds value by enhancing student achievement, facilitating knowledge acquisition, and increasing motivation to learn through features such as alternative representations (geometric, algebraic, various color, font, line, and letter style options), immediate feedback, and a dynamic platform, which together reduce the time and attention required for routine procedures. In line with this, the findings of ^49^ indicated that the traditional paper-and-pencil teaching approach becomes more effective when used alongside technology-assisted instruction in the same classroom.

The post-test results indicate a notable difference in graphing quadratic functions, with the experimental groups performing better than the control groups. The applet enables students to visualize the effects of altering parameters, link concepts with visual aids, and tackle mathematical problems relevant to the secondary school curriculum. ^50^ Noted that low-income countries should focus on curriculum and instruction that incorporate technology. Additionally, their findings revealed that the use of technology significantly enhances students’ motivation and problem-solving skills. Conversely, ^26^ found no relationship between the time spent using technology and the procedural knowledge gained in mathematics learning.

Increased participation and motivation for learning were noted in classrooms that integrated GeoGebra. Supporting this, a study aimed to investigate students’ motivation through the use of technology for teaching transformations, as highlighted by, ^51^ which reached a similar conclusion. Their findings confirm that utilizing GeoGebra significantly enhances students’ learning motivation. Other research, such as, ^52, 53^ also indicated that technology-integrated instruction boosts students’ commitment to learning.

The results of this study indicate that, although GeoGebra-integrated instruction generally has a positive impact on students’ performance, understanding of concepts, and motivation to learn, it is important to note that less impact was seen for certain specific concepts, such as writing the vertex form of a quadratic function, identifying the parameter that shifts the graph of a quadratic function vertically, and defining the axis of symmetry of a parabola.

## Conclusions

The study’s findings have wide-ranging consequences on the effect of alternative instructional approaches for teaching concepts in mathematics. Increased focus on more significant mathematical concepts and decreased effort spent on procedural computations are two benefits of using GeoGebra, as demonstrated by students’ improved achievement. More significantly, GeoGebra may help students comprehend learning concepts by representing them in their preferred ways. Combining these attributes would empower educators to enhance the ways students learn and what they learn. Researchers (for instance, ^50, 54^) have established that when the teaching is assisted by a technology that makes abstract ideas noticeable, teachers can emphasize making connections between concepts, connect abstractions to real-world settings, address robust misconceptions, improve problem-solving abilities, and deal with more advanced or complex ideas appropriate for the students.

This study suggests that a manageable number of computers should be made available to support all students, along with training for teachers on how to effectively use instructional tools like GeoGebra. The roles of teachers and students in the teaching and learning process are notably transformed by technology such as GeoGebra ^1^ Teachers, who incorporate GeoGebra into their classrooms become facilitators, goal-setters, and mentors, guiding students rather than being the sole source of knowledge. Conversely, students take charge of their learning, engage with the tool, and collaborate with their peers, which helps them better understand the concepts being taught compared to traditional classroom settings.

The findings also match up with the social constructivism theory and the ZPD. From a theoretical standpoint, support from colleagues or prompt feedback from teachers on student work can significantly enhance learning. This level of interactivity boosts the overall effectiveness of the educational experience. GeoGebra’s quick feedback and different ways of showing things helped students shift from relying on teachers to learning by fitting the social constructivist view. Therefore, integrating GeoGebra into instruction can make teaching and learning mathematics a more inspiring and resourceful experience for both educators and students. A meta-analysis conducted by ^55^ reviewed five aspects of various studies and found that the effectiveness of GeoGebra software is maximized when used in classrooms with 30 or fewer students, when there are enough computers for individual use (which is crucial for achieving better outcomes), and when the duration of the intervention is four weeks or less. Additionally, while the effectiveness is not dependent on the student’s education level, variations in treatment duration do affect the size of the effect observed.

The result indicates that GeoGebra plays a key role in enhancing students’ understanding of quadratic functions. This not only aids students in grasping essential concepts but also boosts their motivation to learn. The interactive and engaging features of GeoGebra align well with social constructivism principles, fostering an environment conducive to learning. The results suggest that incorporating tools like GeoGebra into mathematics lessons can make challenging concepts more accessible and relatable. These learning interactions lead to increased student engagement, which positively impacts their overall learning experience.

### Limitations of the study

This research provided valuable insights into teaching and understanding mathematical concepts. However, it has some limitations that could influence the results. Firstly, there are several complicating factors to consider, such as the students’ intelligence levels, gender, and the specific nature of the concepts being taught. Secondly, both groups were instructed by the same teachers (the second author), which may have impacted the results. Additionally, the first author consistently guided the teacher on how to implement each activity according to the design. Consequently, these factors could affect the study’s conclusions in various ways. Therefore, further research that incorporates additional confounding variables, such as gender, students’ intelligence levels, and the nature of the concepts, as suggested by, ^56^ is strongly recommended. It is also advisable to conduct a follow-up longitudinal study to assess the durability of the performance and how the motivational effects evolve.

## Data Availability

The data that support the findings of this study are openly available in Zenodo at https://doi.org/10.5281/zenodo.15204573 ^46^ The extended data supporting this study are openly available on Zenodo at https://doi.org/10.5281/zenodo.15565320. ^46^ These materials include:
•
Pre-test_Items.pdf: The set of assessment items administered to students prior to the instructional intervention. •
Teaching_Episodes.pdf: Detailed descriptions of the instructional sessions integrating GeoGebra into the teaching of quadratic functions. •
Post-test_Items.pdf: The set of assessment items administered to students following the instructional intervention. •
Motivation_Questionnaire.pdf: A questionnaire designed to assess students’ motivation and perception towards learning quadratic functions using the GeoGebra integrated instruction. Data are available under the terms of the Creative Commons Attribution 4.0 International license (CC-BY 4.0).
