# Supplementary material for: The effect of GeoGebra integrated instruction on students’ learning of the quadratic function concept
Source: F1000Res. 2025 Jul 7;14:671. [Version 1] doi: 10.12688/f1000research.163113.1 (PMC12645096; doi:10.12688/f1000research.163113.1)
Supplement: Peer review history

# Reviewer report: Reviewer response for version 1

**DOI:** 10.5256/f1000research.179412.r432875

**Published:** 2025-12-11

**Authors:** Irvan Irvan

**Affiliations:** 1 Universitas Muhammadiyah Sumatera Utara, Medan, North Sumatra, Indonesia

Article Summary

 This article investigates the **effect of GeoGebra‑supported mathematics instruction** on **achievement, conceptual understanding, and motivation** of Grade 9 students on the topic of quadratic functions in Ethiopia. The study uses a **quasi‑experimental design** with two nonequivalent groups (control = 45; experimental = 42).

 03aef27b-cbdd-42fe-b8e6-1881fa8…

 Students in the experimental group were taught using **GeoGebra**, whereas the control group was taught using the traditional *chalk‑and‑talk* method. The instruments consisted of a **pre‑test**, a **post‑test**, and a **motivation questionnaire**.

 The results of the study show that:

- There was no initial difference between the two groups (see **Table 2, page 7**).
- There was a significant increase in post‑test scores for the experimental group (see **Tables 3 and 4, pages 8–9**).
- The percentage of correct answers increased across all conceptual outcomes (see **Figures 3–4, pages 10–11**).
- Students’ motivation increased, as shown in **Figure 6, page 11**.

The supporting data, including test items, activity sheets, and questionnaires, are openly available on Zenodo ( **page 14**).

**Full Review Report**

 03aef27b-cbdd-42fe-b8e6-1881fa8…

**A. Major Strengths of the Article**

**1. Presentation and Use of Literature – *Very Good***

 The article is written clearly and references relevant and up‑to‑date literature concerning:

- students’ difficulties in understanding quadratic functions,
- the use of GeoGebra in mathematics instruction, and
- the theoretical foundation of social constructivism (ZPD).

The conceptual explanations and narrative flow are consistent, and the tables and figures effectively support the textual descriptions.

**2. Study Design and Methodological Rigor – *Appropriate and Sound***

 The quasi‑experimental design is appropriate for the educational context in Ethiopia, where random assignment is not always feasible.

 Data collection was systematic, and the instruments were validated by experienced teachers and curriculum experts, then piloted before use.

**3. Statistical Analysis – *Accurate and Well‑Applied***

 The authors used an independent‑samples t‑test, Levene’s test, and calculated effect size (r = 0.83).

 All procedures are explained transparently and meet acceptable statistical standards.

**4. Data Availability – *Excellent***

 All supporting data—including test items, lesson episodes, and questionnaires—are openly available on Zenodo, allowing full reproducibility.

**5. Conclusions and Support from Data – *Strong***

 The conclusions logically follow the data:

- The experimental group scored significantly higher on post‑tests (Table 3 & 4, pp. 8–9).
- Conceptual understanding improved across all outcomes (Figures 3 & 4, pp. 10–11).
- Motivation increased substantially (Figure 6, p. 11).

Thus, findings are robust, consistent, and well‑supported.

**B. Suggestions for Improvement (Minor)**

 These suggestions do *not* undermine the scientific validity of the study but aim to improve clarity and completeness.

**1. Clarify the Application of ZPD (pp. 4–5)**

 Although the four ZPD levels are described, the article would benefit from:

- explicit examples of how each level manifested during instruction,
- clearer articulation of the teacher’s scaffolding role at each stage.

**2. Specify the Classroom Selection Technique (p. 6)**

 The statement *“Students from one classroom in each of the two different schools were selected…”*

 does not explain **how** the classrooms were chosen. The authors should indicate whether the selection was random, purposive, or based on administrative considerations.

**3. Minor Editorial Corrections**

 A few small refinements are recommended:

- Correct the plural form of *syllabus* (p. 6).
- Correct the *were/where* typo (p. 6).
- Check consistency of percentages in **Figure 6** versus textual description.
- Add brief interpretive captions under Tables 2–4 for coherence.

**4. Abstract Improvements**

 The abstract would be more complete if it included:

- the statistical tests used,
- specific recommendations derived from the findings.

**C. Points That *Should* Be Addressed to Strengthen the Article Further**

 These are not major flaws but would enhance the scientific quality of the manuscript:

1. **Clarify the classroom selection procedure** to reduce ambiguity regarding sampling bias.
2. **Double‑check numerical consistency**, especially percentages in Figure 6.
3. **Provide applied examples for the ZPD framework** to enhance theoretical clarity.

These points will strengthen clarity without affecting the study’s validity.

**Overall Review Conclusion**

 This manuscript is **scientifically sound**, methodologically rigorous, and contributes meaningfully to technology‑enhanced mathematics education.

 The study meets the standards for publication. Required improvements are **minor and editorial in nature**.

**Recommendation: Index with Optional Minor Revisions.**

Is the work clearly and accurately presented and does it cite the current literature?

Yes

If applicable, is the statistical analysis and its interpretation appropriate?

Yes

Are all the source data underlying the results available to ensure full reproducibility?

Yes

Is the study design appropriate and is the work technically sound?

Yes

Are the conclusions drawn adequately supported by the results?

Yes

Are sufficient details of methods and analysis provided to allow replication by others?

Yes

Reviewer Expertise:

Mathematics Education

I confirm that I have read this submission and believe that I have an appropriate level of expertise to confirm that it is of an acceptable scientific standard.

# Reviewer report: Reviewer response for version 1

**DOI:** 10.5256/f1000research.179412.r432867

**Published:** 2025-11-24

**Authors:** Torang Siregar

**Affiliations:** 1 Department of Mathematics Education, Faculty of Tarbiyah and Teacher Training (FTIK), UIN Syekh Ali Hasan Ahmad Addary Padangsidimpuan, North Sumatra, Indonesia, Indonesia

**Final Recommendation**

 This study investigates the impact of GeoGebra-integrated instruction on Grade 9 students’ achievement, conceptual understanding, and motivation in learning quadratic functions in Ethiopia. Using a quasi-experimental design with non-equivalent control groups (N = 87; experimental = 42, control = 45), the researchers compared traditional “chalk-and-talk” instruction with GeoGebra-supported teaching over a 10-day intervention. Data were collected via pre- and post-tests and a motivation questionnaire. The theoretical framework draws on Vygotsky’s Social Constructivism and the Zone of Proximal Development (ZPD), positioning GeoGebra as a scaffolding tool. Results indicate statistically significant improvements in the experimental group’s conceptual understanding, problem-solving performance (p < 0.05), and motivation. Effect size was large (r = 0.83), and students reported high satisfaction with the GeoGebra-based approach.

 The article is well-designed, methodologically sound, and provides meaningful evidence regarding the use of GeoGebra in improving mathematical understanding and motivation. With minor clarifications as noted above, the manuscript is suitable for indexing.

**Overall Assessment**

 This is a methodologically robust, transparent, and well-executed study that makes a valuable contribution to technology-enhanced mathematics education, particularly in low-resource contexts. The quasi-experimental design is appropriate, the analysis is statistically sound, and the open sharing of materials ensures reproducibility. The conclusions are fully warranted by the data.

 While the peer reviewer raised minor editorial points (e.g., clarifying ZPD operationalization, correcting wording), none undermine the scientific validity of the work. These are refinements for clarity, not corrections for technical error.

 Recommendation

 ✅ **Accept** as is (with optional minor revisions for clarity per peer reviewer’s suggestions).

 No critical issues require resolution to ensure scientific soundness. The study meets high standards of educational research methodology and reporting.

Is the work clearly and accurately presented and does it cite the current literature?

Yes

If applicable, is the statistical analysis and its interpretation appropriate?

Yes

Are all the source data underlying the results available to ensure full reproducibility?

Yes

Is the study design appropriate and is the work technically sound?

Yes

Are the conclusions drawn adequately supported by the results?

Yes

Are sufficient details of methods and analysis provided to allow replication by others?

Yes

Reviewer Expertise:

Mathematics EducationDigital Learning and Educational TechnologyOpen and Distance Learning (ODL)Technology-Enhanced Learning (TEL)Curriculum Development in MathematicsMathematics Learning EvaluationAssessment for Learning & Assessment as LearningInstructional Design and Learning InnovationSTEM and STEAM EducationProblem-Based LearningMathematical Problem-Solving and Critical ThinkingPedagogical Content Knowledge (PCK)Educational Measurement and Quantitative MethodsClassroom Action ResearchHigher-Order Thinking Skills (HOTS)Teacher Professional Development

I confirm that I have read this submission and believe that I have an appropriate level of expertise to confirm that it is of an acceptable scientific standard.

# Reviewer report: Reviewer response for version 1

**DOI:** 10.5256/f1000research.179412.r397714

**Published:** 2025-08-23

**Authors:** Zutaah Puotier

**Affiliations:** 1 Presbyterian College of Education, Akropong, Ghana

Abstract:

1. The statistical analysis did not include the statistical test used. Consider including the statistical tests. For example, t-test, ANOVA or F-test, etc
2. The abstract did not include recommendations. Consider including the recommendations in the abstract.

Introduction: Ok

 Theoretical Framework:

 The study did not indicate what the ZPD is in this study. By relating the Social Constructivism theory to this study, state the ZPD in the current study. For example, the Social Constructivism theory has some principles.:

 1) Scaffolding

 2) Social community

 3) ZPD, etc.

 By relating the theory to the present study, the use of GeoGebra serves as the scaffolding. Also, consider doing the same for the ZPD. (See page 4 of 16).

 Methodology:

  

1. Students from one classroom in each of the two different schools were selected for the study. How was the selection done? Consider stating the selection technique used. For example, random sampling, purposeful, stratified sampling, etc. (See page 6)
2. Kindly check the plural form of syllabus. (Page 6, in paragraph four (4)).
3. Check this. Is this "were" or "where"? (Page 6, in paragraph five (5)).
4. The Table 2 is a result of pre-test. Consider moving it to the results section. The result of pre-test should appear before the result of the post-test in the results section. (Page 7, in Table two (2)).

Results:

  

1. Nothing is written under Table 2, Table 3, and Table 4. Consider writing something under each Table.
2. Check Figure six, 95.24% of the participants said GeoGebra encourage them to participate in the learning process, while all of them confirmed that the software helped them maintain focused throughout the lesson. (Page 12, in paragraph one (1)).
3. Check Figure six, 95.24% of the participants said it was enjoyable to GeoGebra in the learning process but not "97.62%". (Page 12, in paragraph two (2)).
4. Table 2 presents the Independent sample t-test result of the pre-test but not "Likert scale perception questionnaire". Consider correcting this. (Page 12, in paragraph three (3)).

Discussion : Fine, No changes.

 Conclusion : Fine, No changes.

 Overall évaluation on the paper : Good

 Bibliography/References: Fine, No changes.

Is the work clearly and accurately presented and does it cite the current literature?

Yes

If applicable, is the statistical analysis and its interpretation appropriate?

Yes

Are all the source data underlying the results available to ensure full reproducibility?

Yes

Is the study design appropriate and is the work technically sound?

Yes

Are the conclusions drawn adequately supported by the results?

Yes

Are sufficient details of methods and analysis provided to allow replication by others?

Yes

Reviewer Expertise:

My research interest include technology integration into mathematics education, gender issues in mathematics education, STEM education, inclusivity in mathematics education, Competency based curriculum, and climate change

I confirm that I have read this submission and believe that I have an appropriate level of expertise to confirm that it is of an acceptable scientific standard.

# Response

**Published:** 2025-08-23

**Authors:** Ashebir Sidelil

Dear Zutaah Puotier,

 Thank you very much for your effort in reading deeply and forwarding comments on our article. We will consider all the comments for the final version of the paper.
